# Parke, Davis & Co.

**Published:** 1885-01-20

**Authors:** 


### PARKE, DAVIS & CO.

Parke, Davis & Co., of Detroit, are now conceded to be at the very head of the list of manufacturing Chemists in the United States. In respect to activity, push, enterprise, integrity, and reliability we know of no establishment which surpasses them. They are liberal advertisers, and conduct their business in a fair and honorable basis, and are gentlemanly, courteous and just in all their dealings.

